# *Panax ginseng* Inhibits Metabolism of Diester Alkaloids by Downregulating CYP3A4 Enzyme Activity via the Pregnane X Receptor

**DOI:** 10.1155/2019/3508658

**Published:** 2019-03-21

**Authors:** Liang Yang, Yuguang Wang, Huanhua Xu, Guangyao Huang, Zhaoyan Zhang, Zengchun Ma, Yue Gao

**Affiliations:** ^1^Beijing Institution of Radiation Medicine, Beijing 100850, China; ^2^Anhui Medical University, Hefei 230032, China; ^3^Beijing University of Technology, Beijing 100024, China

## Abstract

To investigate the effects of* P. ginseng* C.A. Mey (*P. ginseng*) on the metabolism of diester alkaloids and explore the potential mechanism. P. ginseng was administered orally to rats for 7 days, after which liver microsome samples were prepared and then incubated with diester alkaloids. Ultra-high performance liquid chromatography-quadrupole time-of-flight mass spectrometry was used to determinate the concentration of diester alkaloids to calculate the clearance rate. The cocktail method was used to evaluate the effects of oral administration of* P. ginseng* extracts on the activities of cytochrome P450 (CYP) isoforms in rats through the changes in the pharmacokinetic parameters of the probe drugs. The protein and gene expression of CYP3A2 and pregnane X receptor (PXR) in rats were evaluated by western blotting and quantitative PCR. The specific enzyme inhibitor method and human recombinant enzyme method were used to identify the involvement of sub-CYPs in the metabolism of diester alkaloids in human liver microsomes (HLMs). The clearances of aconitine, mesaconitine, and hypaconitine in the* P. ginseng* groups were lower than those of the control group. The areas under the curve of midazolam were 2.37 ± 1.05, 4.96 ± 0.51, and 6.23 ± 1.30 mg·L^−1^·h for the low-, medium-, and high-dose* P. ginseng* groups, respectively, which were higher than that of the control (2.23 ± 0.64 mg·L^−1^·h). The clearances of midazolam for the medium- (1.87 ± 0.16 L·h^−1^·kg^−1^) and high-dose (1.60 ± 0.34 L·h^−1^·kg^−1^)* P. ginseng* groups were lower than that of the control group (4.66 ± 1.43 L·h^−1^·kg^−1^). After exposure to* P. ginseng* extracts, the gene and protein expression levels of CYP3A4 and PXR were decreased. The hepatic metabolism rates of aconitine, mesaconitine, and hypaconitine in HLMs were decreased to 60.37%, 21.67%, and 10.11%, respectively, when incubated with ketoconazole, a specific inhibitor for CYP3A. The kinetic plots indicated that the K_M_ and *V*_max_ values of CYP3A4 were 10.08 ± 3.26 *μ*M and 0.12 ± 0.01nmol·mg protein^−1^·min^−1^ for aconitine, 131.3 ± 99.75 *μ*M and 0.73 ± 0.44 nmol·mg protein^−1^·min^−1^ for mesaconitine, and 17.05 ± 9.70 *μ*M and 0.16 ± 0.04 nmol·mg protein^−1^·min^−1^ for hypaconitine, respectively. The in vitro mean intrinsic clearance rates by CYP3A4 were 0.0119, 0.0056, and 0.0091 mL·nmol CYP^−1^·min^−1^ for aconitine, mesaconitine, and hypaconitine, respectively. Therefore we implied that* P. ginseng* inhibited the metabolism of diester alkaloids in vitro and decreased the CYP3A4 enzyme activity as well as the gene and protein expression of CYP3A4 and PXR in vivo. CYP3A4 had a larger effect on diester alkaloid metabolism than the other human CYP isoforms, CYP1A2, CYP2C9, and CYP2E1.

## 1. Introduction

*Panax ginseng* C.A. Mey (*P. ginseng*) and* Radix aconiti lateralis *are used in traditional Chinese medicine. There are several preparations that combine* P. ginseng *and* Radix aconiti lateralis*, such as Shen-fu injection and decoction, which can improve heart failure symptoms and rescue hemorrhagic shock [1–3].

Diester alkaloids are responsible for multiple synergistic toxic effects, such as cardiac toxicity and neurotoxicity in* Radix aconiti lateralis.* Aconitine could cause severe arrhythmias, such as ventricular tachycardia and ventricular fibrillation, by opening membrane sodium channels [4]. The cell membrane integrity impairment caused by the exposure aconitine leads to the efflux of intracellular ionic ([Na^+^], [Ca2^+^], and [K^+^]) and the deactivation of the Na^+^-K^+^-ATPase [5]. Researcher observed the protective effect of the hypaconitine on H9c2 cells under oxygen and glucose deprivation- (OGD-) induced injury, associated with the phosphatidylinositol 3-kinase (PI3K)/Akt signaling pathway [6]. Mesaconitine increased the excitability in rat hippocampal pyramidal cells by an involvement of the noradrenergic system, with inhibition of noradrenaline uptake leading to an enhanced extraneuronal noradrenaline level [7].

Although* Radix aconiti lateralis* is strictly controlled, overdoses have still been reported [8–10]. However, these combined preparations may also increase the risk of toxic accumulation of diester alkaloids. Thus, it is necessary to understand whether the metabolism of diester alkaloids is affected by* P. ginseng* and explore the potential mechanism. In our previous study, we had suggested that* P. ginseng *could inhibit the metabolism of diester alkaloids in rats, but the mechanism was still unclear.

Most interactions related to pharmacokinetics involve drug-metabolizing cytochrome P450 (CYP) enzymes [11–14]. CYP enzymes, which are the main metabolic enzymes involved in phase I metabolism, participate in the metabolism of most endogenous and exogenous substances. There are several CYP enzyme isoforms, including CYP1A2, CYP2B, CYP3A4 (CYP3A2 in rats), CYP2C9 (CYP2C11 in rats), and CYP2E1. CYP3A4 accounts for 60% of all these subtypes. CYP3A has ethnic diversity; the function of CYP3A2 in rats is similar to that of CYP3A4 in human, so researchers often evaluate the effect of drugs on CYP3A2 in rats to predict its possible effect on CYP3A4 in human [15–18]. Nuclear receptor superfamily (NRs) has been identified as playing a pivotal role in signal transduction for many xenobiotics. CYP enzyme activities are regulated by NRs, which can increase the efficacy or reduce the toxicity of exogenous substances. Among them, aryl hydrocarbon receptor (AhR) regulates the expression of CYP1A1[19, 20], constitutive androstane receptor (CAR) regulates CYP2B6 [21], and pregnane X receptor (PXR) regulates CYP3A4. PXR exerts CYP3A4 transcriptional regulation by binding to its DNA response elements as a heterodimer with the retinoid X receptor (RXR) and recruitment of a host of coactivators [22].

The components of* P. ginseng* may regulate CYP3A4 enzyme activity via PXR [23, 24]. However, most studies of the effects of* P. ginseng* on CYP enzyme activity have focused on single compounds or* in vitro *experiments [23–30], and the combined effects of* P. ginseng* compounds on CYPs and PXR have not been reported. Moreover, specific CYP isoforms screened for the metabolism of diester alkaloids have not been fully elucidated.

In this study, the clearances of diester alkaloids in liver microsomes obtained from rats exposed to* P. ginseng* were calculated. The cocktail method was used to evaluate the effects of* P. ginseng* on the in vivo activities of CYP enzyme isoforms CYP1A2, CYP2C9, CYP2E1, and CYP3A4, which were reflected by the changes in the pharmacokinetic parameters of the probe drugs caffeine, tolbutamide, chlorzoxazone, and midazolam, respectively. Furthermore, specific CYP inhibitors alpha-naphthoflavone (ANF; CYP1A2), sulfaphenazole (SPE; CYP2C9), diethyldithiocarbonate (DDTC; CYP2E1), and ketoconazole (KEN; CYP3A4) in human liver microsomes (HLMs) and recombinant human cytochrome P450 enzymes (including CYP1A2, CYP2C9, CYP2E1, and CYP3A4) were used to identify CYP enzyme isoforms that metabolize diester alkaloids.

## 2. Materials and Methods

### 2.1. Preparation of* P. ginseng* Extract

*P. ginseng *(300 g, Qiancao Group Co., Ltd., Beijing, China; produced in Jilin Province, China) was soaked in 2.4 L double-distilled H_2_O for 30 min and boiled for 1 h, and then the extract was filtered. The roots were reextracted with 1.8 L double-distilled H_2_O for 30 min, and then the extract was filtered. The mixture was concentrated under vacuum to 300 mL. The 0.1 g/L* P. ginseng* extract was centrifuged at 12,000 ×* g* for 15 min, and the supernatant was transferred into another centrifugation tube, following by filtering with a 0.22-*μ*m micropore membrane. The supernatant (5 *μ*L) was injected into an ultra-high performance liquid chromatography-quadrupole time-of-flight mass spectrometry (UPLC-Q/TOF-MS) system for analysis.

### 2.2. Study Animals and Treatments

Male Sprague-Dawley rats weighing 100–140 g were obtained from the Animal Experiment Center, Academy of Military Medical Science of the Chinese People's Liberation Army (license: SCXK army-2012-0004; Beijing, China). The animals were housed in a controlled environment, with a room temperature of 22 ± 2°C, humidity of 50 ± 5%, and a 12-h dark/light cycle. All animals were kept in this environment for 3 days before the experiments. Animal experiments were performed in accordance with the European Commission guidelines. This research was approved by the ethics review board of Animal Care and Use Committee, Academy of Military Medical Science of the Chinese People's Liberation Army (approval number: IACUC-AMS-13-2017-010). All animals were anesthetized using isoflurane and euthanized by carbon dioxide inhalation to minimize suffering.

Thirty rats were randomly divided into the following five groups: the control group,* P. ginseng low* group ( Ginseng-L),* P. ginseng *medium group ( Ginseng-M),* P. ginseng* high group( Ginseng-H), and the KEN group. In the control group, the rats were administered physiological saline at a dose of 4 mg·kg^−1^. In the* P. ginseng* groups, rats were orally administered* P. ginseng* extracts at doses of 1, 5, or 10 mg·kg^−1^ for the low-, medium-, and high-dose groups, respectively, once daily, for 7 consecutive days. In the KEN group, rats were administered KEN (CAS: 65277-42-1; EFEBIO Co., Ltd., Shanghai, China) at a dose of 40 mg·kg^−1^ through intraperitoneal injection, once daily, on 3 consecutive days before the probes were administered. On the 8^th^ day, the probe drugs, consisting of caffeine (2.5 mg·kg^−1^; CAS:58-08-2), chlorzoxazone (5 mg·kg^−1^; CAS:95-25-0), tolbutamide (2.5 mg·kg^−1^; CAS:64-77-7), and midazolam (10 mg·kg^−1^; CAS:59467-70-8; all from Sigma-Aldrich, St. Louis, MO, USA), were administered to all groups through the caudal vein. About 0.5 mL plasma was collected in 1.5-mL centrifuge tubes containing heparin sodium at 5, 15, 30, 45, 60, 90, 120, 240, 480, 720, and 1440 min. After centrifugation at 3,500 ×* g* for 15 min, the supernatant was decanted into another centrifuge tube and stored at -20°C. Liver tissue was washed with physiological saline and stored at -80°C.

### 2.3. Preparation of Rats Plasma Samples

Liquid-liquid extraction was used to prepare the plasma samples. The plasma (200 *μ*L) was spiked with methanol (800 *μ*L) and diazepam (20 *μ*L, 50 *μ*g·mL^−1^; CAS: 439-14-5; Kingyork Group Co., Ltd., Tianjin, China). After vortexing for 15 s and centrifugation at 12,000 ×* g* for 15 min, the supernatant was transferred into a 1.5-mL centrifuge tube and dried under a nitrogen stream at 37°C. The residue was reconstituted in 100 *μ*L acetonitrile/water (1:1, v/v), centrifuged at 12,000 ×* g* for 15 min, and then filtered with a 0.22-*μ*m micropore membrane. The supernatant (5 *μ*L) was injected into the UPLC-Q/TOF-MS system for analysis.

### 2.4. Effects of* P. ginseng* on the Metabolism of Aconitine, Mesaconitine, and Hypaconitine

Preparation of rat liver microsomes: the rats were anaesthetized and perfused with precooled saline. The liver was extracted, and part (5 g) of the liver tissue was treated with TMS [20 mL; Tris-base (12.11 g), MgCl_2_·6H_2_O (1.21 g), and sucrose (137 g) in water (1 L)] on ice. After centrifugation at 12,000 ×* g* at 4°C for 20 min and at 10,500 ×* g* at 4°C for 60 min, the sediment was treated with Tris-HCl [5 mL; Tris-base (12.12 g) and EDTA-2Na (0.37 g) in water (800 mL) and glycerol (200 mL)]. Total protein concentration was determined by a bicinchoninic acid assay (CW Biotech Co., Ltd., Beijing, China), and the samples were stored at -80°C.

A rat liver microsome incubation system was used to detect the clearance of aconitine, mesaconitine, and hypaconitine. Rat liver microsome were treated with 2 mM MgCl_2_ and 2 mM NADPH containing 15 *μ*M aconitine (CAS: 302-27-2, EFEBIO Co., Ltd.), 15 *μ*M mesaconitine (CAS: 2752-64-9, EFEBIO Co., Ltd.), or 15 *μ*M hypaconitine (CAS: 6900-87-4, EFEBIO Co., Ltd.) according to the experimental requirement. The mixtures were incubated at 37°C for 60 min in water bath kettle. NADPH was preincubated for 5 min before it was added to the system. Precooled acetonitrile/methanol 800 *μ*L (1:1, v/v) was added to stop the incubation. Preparation of samples were refer to plasma preparation of plasma samples above.

### 2.5. Identification of CYP Isoforms for Aconitine, Mesaconitine, and Hypaconitine Metabolism

To screen the CYP isoforms for aconitine, mesaconitine, and hypaconitine metabolism, we used known inhibitors of the metabolism of aconitine, mesaconitine, and hypaconitine in HLMs [31]. ANF (CYP1A2: 1 *μ*M; CAS: 604059-1), SPE (CYP2C9: 1 *μ*M; CAS: 526-08-9), DDTC (CYP2E1: 1 *μ*M; CAS: 148-18-5), and KEN (CYP3A4: 1 *μ*M; CAS: 65277-42-1) were all from EFEBIO Co., Ltd., which were incubated separately with aconitine, mesaconitine, and hypaconitine (20 *μ*M), HLMs, and NADPH under the same incubation conditions as mentioned above. The inhibitory effects of the inhibitors on the hepatic metabolism rate of aconitine, mesaconitine, and hypaconitine were evaluated separately to identify the CYP isoforms responsible for their metabolism. The hepatic metabolism rate of the incubation solution with no inhibitor was used as the negative control. The HLM (Rild-biotech, shanghai, China, X008067 ) mixtures were incubated at 37°C for 60 min. NADPH was preincubated for 5 min before it was added to the system. Precooled acetonitrile/methanol (800 *μ*L, 1:1, v/v) was added to stop the incubation. After vortexing for 15 s and centrifugation at 1,2000g and 4°C for 15 min, the supernatant was transferred into another 1.5-mL centrifuge tube and dried under a nitrogen stream at 37°C. The residue was reconstituted in 100 *μ*L acetonitrile/water (1:1, v/v) and was centrifuged at 14,000 for 15 min. The sample was filtered with a 0.22 *μ*m micropore membrane and the supernatant (5 *μ*L) was injected into the UPLC-Q/TOF-MS system for analysis. Aconitine, mesaconitine, and hypaconitine standards of different concentrations were added to the incubation system to obtain the standard curve.

### 2.6. Kinetic Analysis of Aconitine, Mesaconitine, and Hypaconitine in Recombinant Human Cytochrome P450 Enzymes

Recombinant human cytochrome P450 enzymes were obtained from Corning (USA, CYP3A4:456202; CYP1A2:456203; CYP2C9:456218; CYP2E1:456206). The activity were catalyzed by CYP3A4/CYP1A2/CYP2C9/CYP2E1, which were expressed from human CYP3A4/CYP1A2/CYP2C9/CYP2E1 cDNA, respectively, using a baculovirus expression system. Baculovirus infected insect cells (BTI-TN-5B1-4) were used to prepare these microsomes. These microsomes also contain cDNA-expressed humans P450 reductase and human cytochrome b5. A microsome preparation using wild type virus (Catalog No. 456201) was used as a control for native activities.

The kinetic parameters for the metabolism of aconitine, mesaconitine, and hypaconitine were determined by incubating increasing concentrations of aconitine, mesaconitine, and hypaconitine (5, 10, 20, and 40 *μ*M) at 37°C for 90min with recombinant human CYP isoforms and NADPH under the incubation conditions described above [32]. Precooled acetonitrile/methanol (800 *μ*L, 1:1, v/v) was added to stop the incubation. After vortexing for 15 s and centrifugation at 1,2000g and 4°C for 15 min, the supernatant was transferred into another 1.5-mL centrifuge tube and dried under a nitrogen stream at 37°C. The residue was reconstituted in 100 *μ*L acetonitrile/water (1:1, v/v) and was centrifuged at 14,000 for 15 min. The sample was filtered with a 0.22 *μ*m micropore membrane and the supernatant (5 *μ*L) was injected into the UPLC-Q/TOF-MS system for analysis.

The compound reaction velocity (*V*) is expressed as* V* = (*C*_0_ – *C*_t_)/*T*/*C*_p_, where* C*_0_ and *C*_t_ are the initial and final concentrations of aconitine, mesaconitine, or hypaconitine in the incubation solution, respectively,* T* is the incubation time (min), and *C*_p_ is the protein concentration (mg·mL^−1^ or nmol). The mean intrinsic clearance rate (CL_int_) for the in vitro incubation was estimated using *V*_max_/*K*_*M*_, where *V*_max_ is the maximum velocity and K_M_ is the Michaelis constant. All values are expressed as the mean ± standard deviation (SD).

### 2.7. UPLC-Q/TOF-MS Analysis

All samples were analyzed with a Aquity UPLC-Q/TOF-MS system (Waters Synapt G1, Milford, MA, USA). Chromatographic separation was conducted on the Waters ACQUITY UPLC HSS T3 1.8-*μ*m column (2.1 × 100 mm) at 40°C and a flow rate of 0.5 mL·min^−1^.

Phase A was water and 0.1% formic acid (v/v), and phase B was 100% acetonitrile. The gradient elution programs for P. ginseng extract are as follows: 0 min, 2% B; 1 min, 20% B; 27 min, 50% B; and 29–30 min, 2% B. The gradient elution programs for rats plasma samples are as follows: 0 min, 2% B; 1 min, 12% B; 2–4 min, 25% B; 6–8 min, 28% B; 10–12 min, 29% B; 13–14 min, 29.5% B; 15–16 min, 30% B; 17–18 min, 35%–100% B; and 19–20 min, 2% B. The gradient elution programs for rat liver microsomes and HLMs are as follows: 0 min, 2% B; 1 min, 25% B; 2 min, 30% B; 6 min, 40% B; and 9–10 min, 2% B.

Electrospray ionization and negative ion V mode were used to analyze the* P. ginseng* extracts and chlorzoxazone, and other compounds were acquired in positive ion V mode. Quantification for detecting the probe drugs was performed using full scan mode. The main parameters for the mass spectroscopy were as follows:* m*/*z*, 100–1000; capillary voltage, 3 kV; cone voltage, 40 V; ion source temperature, 100°C; desolvation temperature, 450°C; injection volume, 5 *μ*L; and desolvation rate, 900 L·h^−1^. Leucine enkephalin was used to correct the molecular weight ([M - H]^−^, 554.2615; [M + H]^+^, 556.2771).

### 2.8. Western Blot Analysis

For western blotting, equal amounts of proteins were resolved by 10% SDS-PAGE (CW Biotech Co., Ltd.). Proteins were blotted onto a methanol-charged PVDF membrane using a wet transfer system (70 V, 2 h). The membrane was blocked with 5% bovine serum albumin for 1.5 h at room temperature, washed with Tris-buffer saline containing 0.1% Tween-20 (TBST) three times (5 min each time), and then incubated with the primary antibodies anti-PXR (1:1000, ab192579, Abcam, Cambridge, UK), anti-CYP3A2 (1:1000, ab195627, Abcam), or anti-GAPDH (1:4000, ab94282, Abcam). The membrane was washed with TBST five times (5 min each) and incubated for 1 h with goat anti-rabbit IgG H&L (HRP) secondary antibody (1:5000, ab6721, Abcam). After five washes with TBST, the protein bands were detected by chemiluminescence reagents (ECL kit ab133406, Abcam). An imaging system (ImageQuant LAS 500, GE Heathcare Life Sciences, Chicago, IL, USA) was used to visualize the protein bands, and densitometry was performed with Image J software. The density of each immunoreactive band was normalized to the density of its corresponding GAPDH band.

### 2.9. Quantitative PCR

Quantitative PCR was used to evaluate the gene expression of CYP3A4 and PXR. Equal amounts of total RNA were conducted by TRIzol (Sigma-Aldrich), and reverse transcriptional and quantitative PCR were conducted according to the manufacturer's instructions. RNA was reverse transcribed (Trans Gen, Beijing, China) and then used for quantitative real-time PCR (Applied Biosystems StepOnePlus™, Foster City, CA, USA). Primers were synthesized by Sangon Biotech (Sangon, Shanghai, China). The primer sequences for quantitative real-time PCR are provided in the data supplement (Table S1).

### 2.10. Statistical Analysis

Data were expressed as the mean ± SD, which were calculated and analyzed by Mass Lynx 4.1 software (Waters). The pharmacokinetic parameters were analyzed by DAS Version 2.0. SAS statistical software was used to determine the group differences. Figures were processed and displayed with GraphPad Prism Version 5.01. The theoretical and measured values for the molecular weights of relevant compounds from the* P. ginseng* extract were compared, and the relative standard deviation (RSD) was calculated.* P* < 0.05 was considered statistically significant.

## 3. Results

### 3.1. *P. ginseng *Inhibits Clearance of Aconitine, Mesaconitine, and Hypaconitine

**Figure 1:**
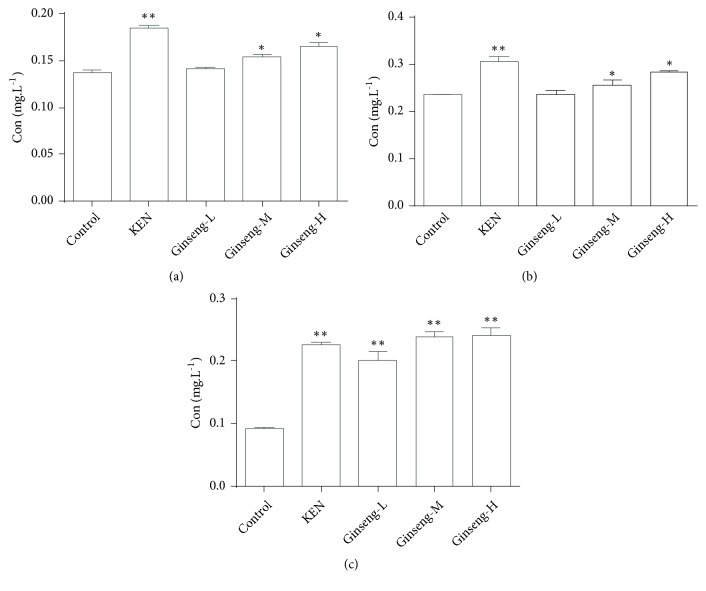
Concentrations of aconitine (a), mesaconitine (b), and hypaconitine (c) in the liver microsome incubation system after oral administration of* P. ginseng*. Compared with the control group, ^*∗*^*P* < 0.05; ^*∗∗*^*P* < 0.01 (*n* = 3). Ginseng-L:* P. ginseng* low group; Ginseng-M:* P. ginseng* middle group; Ginseng-H:* P. ginseng* high group.

To investigate whether the metabolism of aconitine, mesaconitine, and hypaconitine was affected by* P. ginseng*, the contents of aconitine, mesaconitine, and hypaconitine were determined in the incubation system after exposure to* P. ginseng*. The concentrations of aconitine, mesaconitine, and hypaconitine were higher in the* P. ginseng* groups compared with the control group (Figure 1). The standard curves of aconitine, mesaconitine, and hypaconitine in the biological samples are shown in Table S2.

### 3.2. Effects of* P. ginseng* on CYP Isoform Activities in Rats

The cocktail method was used to evaluate the effects of* P. ginseng* on CYP isoform activities in rats. The probe drugs, caffeine, midazolam, tolbutamide, and chlorzoxazone, were validated as follows. The chromatograms of various probe drugs in plasma samples were shown in Figure S1. The standard curve, linearity, and correlation coefficient of each component in the selected concentration range are shown in Table S3. All the correlation coefficients were larger than 0.9900. Furthermore, the intraday and interday precision and accuracy of the probe drugs were determined in the linear range (Table S4). Three different quality control concentrations were analyzed for each probe: caffeine, 0.1, 5, and 50 *μ*g·mL^−1^; midazolam, 0.024, 0.12, and 12 *μ*g·mL^−1^; tolbutamide, 5, 25, and 50 *μ*g·mL^−1^; and chlorzoxazone, 0.1, 5, and 50 *μ*g·mL^−1^. The average intraday and interday precision and accuracy for the high, medium, and low concentrations ranged from 97.60% to 110.96%. The RSDs of intraday and interday precision and accuracy were less than 10%. Our results showed that the accuracy of all the probe drugs was less than 15%. The stability of the related temperature is shown in Table S5-1, and the freeze-thaw stability is shown in Table S5-2. The recoveries of the methods for the four probe drugs were from 91.41% to 102.11%, and the RSDs were from 2.48% to 10.36% (Table S6).

**Figure 2:**
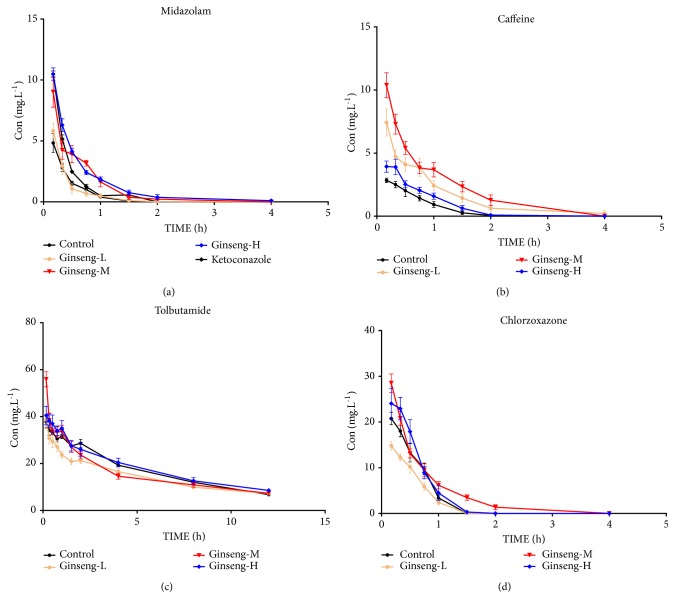
Blood concentration-time curves of probe drugs after administration of* P. ginseng*. Blood concentration-time curves of midazolam (a), caffeine (b), tolbutamide (c), and chlorzoxazone (d) of rats after oral administration of* P. ginseng* for 7 days. Ginseng-L:* P. ginseng* low group; Ginseng-M:* P. ginseng* middle group; Ginseng-H:* P. ginseng* high group.

The blood concentration-time curve of midazolam is shown in Figure 2(a) and the pharmacokinetic parameters of midazolam are shown in Table 1. The area under the curve (AUC(0-t)) values of midazolam in the low-, medium-, and high-dose P. ginseng groups were higher than that of the control group. The clearance (CLz) of midazolam in the medium-, and high-dose P. ginseng groups was significantly lower than that of the control (P < 0.01). The blood concentration-time curve of caffeine is shown in Figure 2(b). The pharmacokinetic parameters of caffeine for each group are shown in Table 2. Compared with the control group, the CL_z_ values of caffeine in the low-, medium-, and high-dose* P. ginseng* groups were lower, whereas the AUC_(0-t)_, AUC_(0-*∞*)_, and *C*_max_ values were higher. The blood concentration-time curves of tolbutamide and chlorzoxazone are shown in Figures 2(c) and 2(d). The blood pharmacokinetic parameters of tolbutamide are shown in Table 3. There were no significant differences between the AUC_(0-t)_ and CL_z_ values for tolbutamide in the* P. ginseng* groups compared with the control group. The blood pharmacokinetic parameters of chlorzoxazone are shown in Table 4. The AUC_(0-t)_ values of chlorzoxazone in the medium-dose (19.05 ± 4.28 mg·L^−1^·h)* P. ginseng* group were higher than that of the control (13.53 ± 2.42 mg·L^−1^·h), but the AUC_(0-t)_ value in the high-dose group (16.72 ± 5.96 mg·L^−1^·h) had no significant difference compared with the control.

### 3.3. Effects of P. ginseng Extracts on PXR and CYP3A2 RNA and Protein Expression in Rats

**Figure 3:**
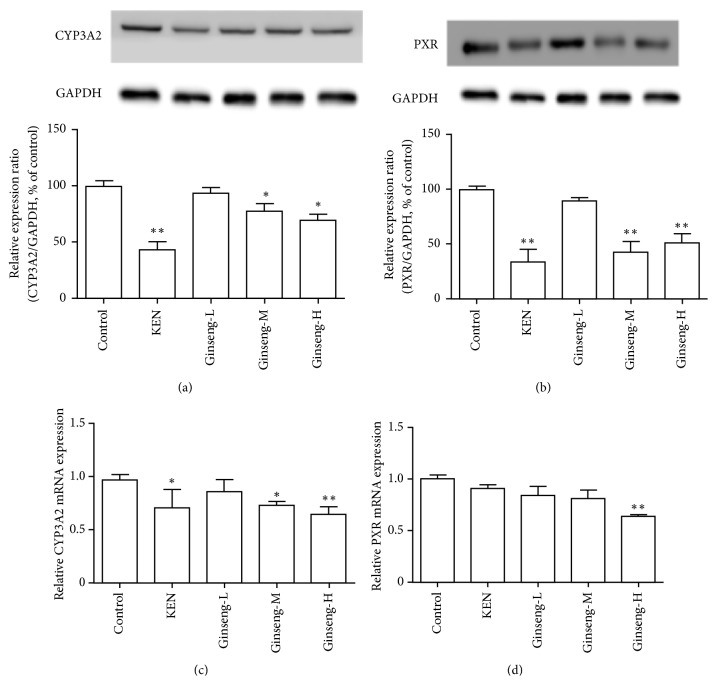
Mediation by PXR of CYP3A2 downregulation by* P. ginseng*. Effects of* P. ginseng* on CYP3A2 (a) and PXR (b) protein expression in Sprague-Dawley rats. KEN was used as a negative control. Effects of* P. ginseng* on CYP3A2 (c) and PXR (d) mRNA expression in Sprague-Dawley rats. Data represent mean ± SD (*n* = 3). Compared with the control group, ^*∗*^*P* < 0.05; ^*∗∗*^*P* < 0.01.

The CYP3A2 (isozyme of CYP3A4) and PXR protein and gene expression levels in Sprague-Dawley rats exposed to* P. ginseng* extracts were evaluated by western blotting and quantitative PCR. Compared with the control groups,* P. ginseng* downregulated the expression of CYP3A2 and PXR at both the protein (Figures 3(a) and 3(b)) and gene levels (Figures 3(c) and 3(d)).

### 3.4. Inhibition of CYP3A Enzyme Activity Decreases the Clearance of Aconitine, Mesaconitine, and Hypaconitine

**Figure 4:**
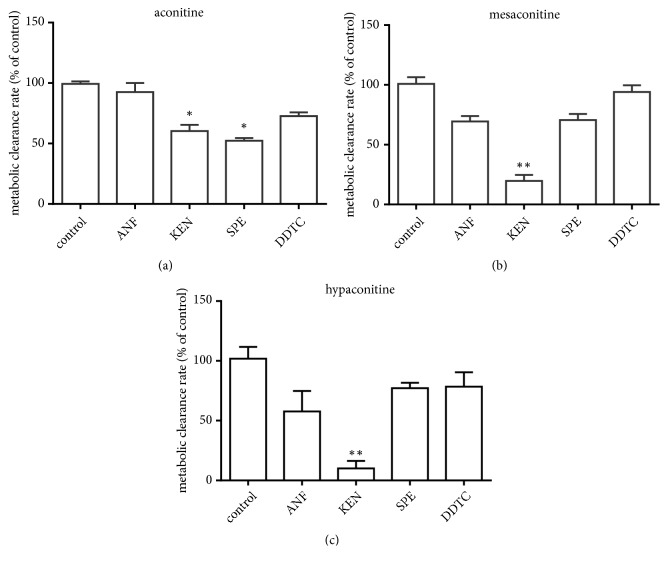
Effect of specific inhibitors KEN, ANF, SPE, and DDTC on CYP-mediated aconitine (a), mesaconitine (b), and hypaconitine (c) metabolism in HLMs (*n* = 3). Compared with the control group, ^*∗*^*P* < 0.05; ^*∗∗*^*P* < 0.01.

The inhibitory effects of the CYP-specific inhibitors on the hepatic metabolism rates of aconitine, mesaconitine, and hypaconitine in the HLMs are shown in Figure 4. The hepatic metabolism rates of aconitine, mesaconitine, and hypaconitine when incubated with KEN (1 *μ*M, specific inhibitor for CYP3A) decreased to 60.37 ± 5.01%, 21.67 ± 19.64%, and 10.11 ± 6.19%, respectively, compared with the negative controls. After incubation with SPE, the hepatic metabolism rate of aconitine decreased to 52.25 ± 2.26%, compared with the negative control. However, after incubation with ANF and DDTC, the hepatic metabolism rates of aconitine, mesaconitine, and hypaconitine showed no significant change compared with the control (*P* > 0.05).

### 3.5. Kinetic Analysis of Aconitine, Mesaconitine, and Hypaconitine in Recombinant Human Cytochrome P450 Enzymes System

**Figure 5:**
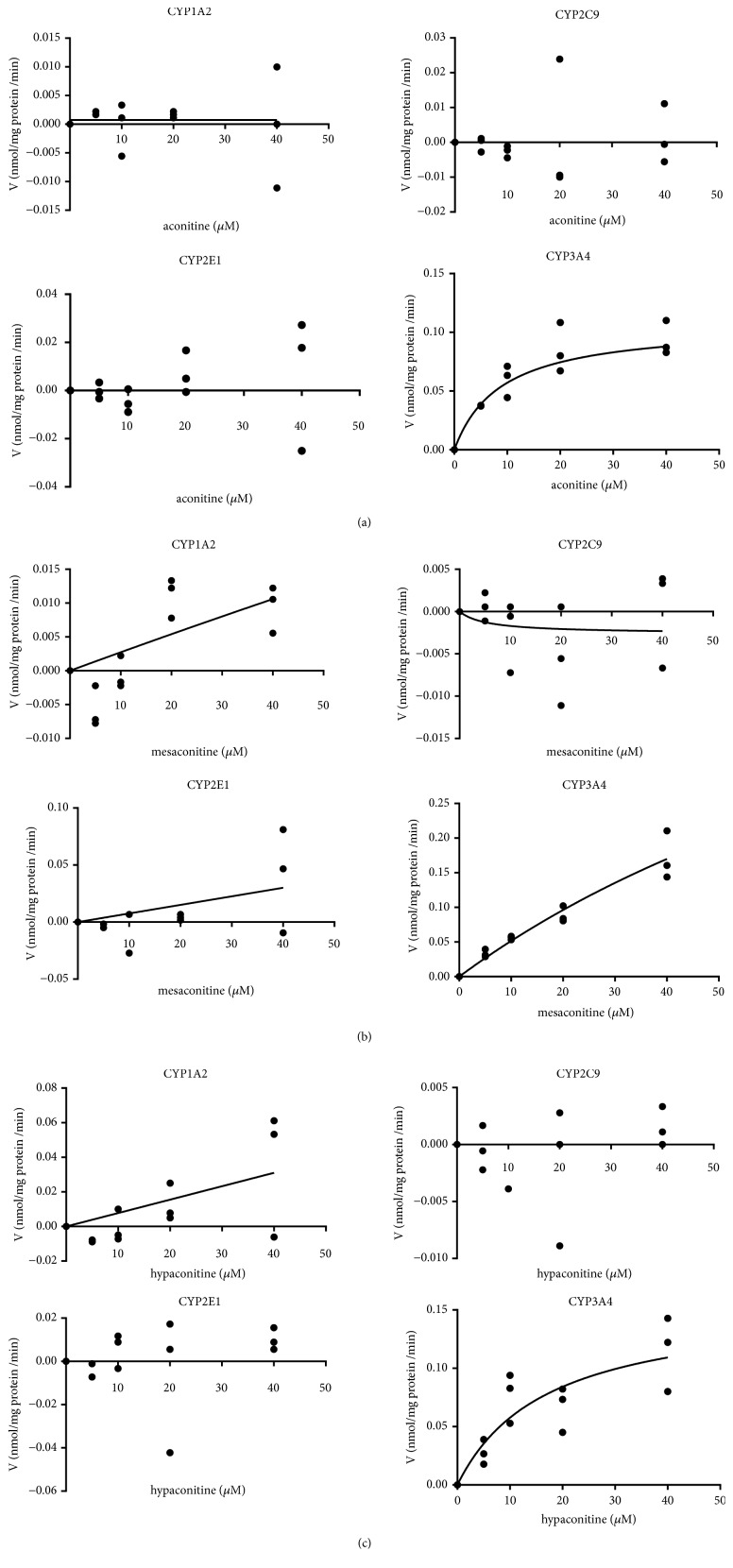
Concentration-velocity curves of aconitine (a), mesaconitine (b), and hypaconitine (c) metabolism after incubation with recombinant human cytochrome P450 enzymes (*n* = 3). The incubation conditions are described in the Materials and Methods section. The curve was automatically fitted using nonlinear regression and the Michaelis–Menten equation.

Figure 5 shows the metabolism of aconitine, mesaconitine, and hypaconitine after incubation with recombinant human CYP3A4, CYP1A2, CYP2C9, and CYP2E1. The kinetic plots indicated that the K_M_ and *V*_max_ values of CYP3A4 were 10.08 ± 3.26 *μ*M and 0.12 ± 0.01nmol·mg protein^−1^·min^−1^ for aconitine, 131.3 ± 99.75 *μ*M and 0.73 ± 0.44 nmol·mg protein^−1^·min^−1^ for mesaconitine, and 17.05 ± 9.70 *μ*M and 0.16 ± 0.04 nmol·mg protein^−1^·min^−1^ for hypaconitine, respectively. The kinetic plots indicated that the K_M_ and *V*_max_ values of CYP1A2 were 1.624e^−16^  ± 0.00 *μ*M and 7.407e^−4^  ±0.00 nmol·nmol protein^−1^·min^−1^, 981.6 ± 24664 *μ*M and 0.27 ± 6.57 nmol·mg protein^−1^·min^−1^for mesaconitine, and 4.191e^17^  ±7.180e^32^*μ*M and 3.250e^14^  ± 5.569e^29^ nmol·nmol protein^−1^·min^−1^for hypaconitine. The kinetic plots indicated that the K_M_ and *V*_max_ values of CYP2E1 were 1.752e^19^± 3.593e^12^*μ*M and 1.318e^16^  ±4.775e^15^ nmol·nmol protein^−1^·min^−1^ for mesaconitine.

The in vitro mean intrinsic clearance rates by CYP3A4 were 0.0119, 0.0056, and 0.0091 mL·nmol CYP^−1^·min^−1^ for aconitine, mesaconitine, and hypaconitine, respectively.

## 4. Discussion

Small changes in the concentrations of diester alkaloids could have a significant effect on the toxicity and efficacy of* Radix aconiti lateralis *[7, 8]. Due to the toxicity of diester alkaloids, the dosages of commercially available drugs containing* Radix aconiti lateralis* are strictly limited in the Chinese Pharmacopoeia (2015). Our results showed that the concentrations of aconitine, mesaconitine, and hypaconitine in the* P. ginseng* group were higher than in the control group (Figure 1), which suggested that* P. ginseng* inhibits the metabolism of aconitine, mesaconitine, and hypaconitine. As the key toxic and effective components in Aconiti Lateralis Radix, the concentration of DAs in vivo is strictly controlled. In our previous study, we had suggested that P. ginseng can inhibit the clearance of aconitine, mesaconitine, and hypaconitine in rats. This results in the fact that the accumulation of aconitine, mesaconitine, and hypaconitine within the body may be the basis of DDI between P. ginseng and Aconiti Lateralis Radix [33].

CYPs are the main type of metabolic enzyme in phase I metabolism and they participate in the metabolism of most endogenous and exogenous substances [15]. We evaluated the effects of* P. ginseng* on CYP activities by using the cocktail method. A refined method was proposed using the probe drugs, caffeine, midazolam, tolbutamide, and chlorzoxazone. In preliminary experiments, testosterone was chosen as the probe drug for CYP3A4. However, considering the stability and interference from endogenous testosterone, midazolam was eventually selected as the probe drug for CYP3A4. The method had good precision, accuracy, and stability, and the recoveries of the probe drugs in the plasma samples were from 91.41 ± 8.61% to 102.11 ± 6.89% (Table S6). CYP3A4 (Table 1) and CYP1A2 (Table 2) activities were inhibited by* P. ginseng *in vivo (Figures 2 and 3). Synergistic activation or antagonism effects are common in traditional Chinese medicine. PXR regulates the expression of the CYP subunit, which is central to defending the body from toxic substances, and thus PXR is crucial in increasing the efficacy or reducing the toxicity of active compounds [17, 18]. Our results implied that PXR was significantly inhibited by oral administration of* P. ginseng *in rats (Figure 3). It is also suggested that there is no dose-dependent relationship between the effects of P. ginseng on PXR protein expression. This is probably because different components of P. ginseng had different effects on PXR, such as ginsenoside F2 and protopanaxatriol had moderate inductive effects on PXR, while Panaxotriol, ginsenoside Rg2, pseudoginsenoside F11, ginsenoside Rg1, and ginsenoside Rb3 had inhibitory effects on PXR. The non-dose dependence is the result of the complex interaction of the monomer components [26].

CYP450 enzymes, which are the main metabolic enzymes involved in phase I metabolism, participate in the metabolism of most endogenous and exogenous substances. Researchers found that CYP450 enzymes involved in aconitine metabolism in vitro, hydroxylation, di-demethylation, and dehydrogenation were conducted by human recombinant CYP450. Aconitine can be transformed into at least six CYP-mediated metabolites in HLMs; CYP 3A4/5 and 2D6 were the most important CYP isoforms responsible for the de-methylation, N-deethylation, dehydrogenation, and hydroxylation of aconitine [32]. But CYP isoforms responsible for the metabolism of mesaconitine and hypaconitine are still unclear. Here, CYP-specific inhibitors and a recombinant human cytochrome P450 enzymes system were used to screen CYP isoforms for metabolism of aconitine, mesaconitine, and hypaconitine. The hepatic metabolism rate of aconitine, mesaconitine, and hypaconitine when incubated with KEN was significantly decreased. However, incubation with ANF, SPE, and DDTC did not alter the hepatic metabolism rates of aconitine and mesaconitine, and ANF also decreased the hepatic metabolism rate of hypaconitine (Figure 4). Compared with hCYP1A2, hCYP2C9, and hCYP2E1, incubation with hCYP3A4 significantly decreased K_M_ and *V*_max_ and increased CL_int_ of aconitine and mesaconitine. For incubation with hCYP1A2, K_M_ and *V*_max_ of hypaconitine decreased and CL_int_ increased (Figure 5). Thus, CYP3A4 was the major sub-CYP for the metabolism of aconitine and mesaconitine, and both CYP3A4 and CYP1A2 were important in the metabolism of hypaconitine.

In our previous work, we found that the components of* P. ginseng* had different effects on PXR. Ginsenoside F2 and protopanaxatriol had moderate inductive effects on PXR. Panaxotriol, ginsenoside Rg2, pseudoginsenoside F11, ginsenoside Rg1, and ginsenoside Rb3 had inhibitory effects on PXR, whereas ginsenoside Rf1, Rg3, Rh2, and protopanaxadiol had no obvious effects on PXR. Ginsenoside Rg1 downregulated CYP3A4 mRNA expression in a concentration-dependent manner [28]. The combined effects of P. ginseng extracts on CYP3A4 and PXR had more practical significance than the effects of monomer components on CYP3A4 and PXR, because it is more close to clinical use. This work is a supplement for the total extracts from* P. ginseng *on the CYPs activities and PXR expression in vivo.

## 5. Conclusion

*P. ginseng* inhibited the metabolism of diester alkaloids in vitro.* P. ginseng* decreased CYP3A4 enzyme activity in vivo, as well as the gene and protein expression of CYP3A4 and PXR. CYP3A4 showed a greater effect on diester alkaloid metabolism than other human CYP isoforms, including CYP1A2, CYP2C9, and CYP2E1.

## Figures and Tables

**Table 1:** Effect of *P. ginseng* on the pharmacokinetics of midazolam at day 7 after administration.

Parameters/unit	Control	Ketoconazole	*P. ginseng*-L	*P. ginseng*-M	*P. ginseng*-H
AUC_(0-t)_/mg^.^L^-1.^h	2.23 ± 0.64	4.19 ± 0.14^*∗∗*^	2.37 ± 1.05	4.96 ± 0.51^*∗∗*^	6.23 ± 1.30^*∗∗*^
AUC_(0-*∞*)_/mg^.^L^-1.^h	2.30 ± 0.59	4.25 ± 0.15^*∗∗*^	2.42 ± 1.06	5.38 ± 0.44^*∗∗*^	6.58 ± 1.79^*∗∗*^
t_1/2z_/h	0.30 ± 0.15	0.24 ± 0.06	0.25 ± 0.12	0.38 ± 0.30	0.56 ± 0.52
T_max_/mg^.^L^−1^	0.11 ± 0.07	0.08 ± 0.00	0.08 ± 0.00	0.11 ± 0.07	0.08 ± 0.00
CL_z_/L^.^h^-1.^kg^−1^	4.66 ± 1.43	2.36 ± 0.08^*∗∗*^	5.07 ± 2.74	1.87 ± 0.16^*∗∗*^	1.60 ± 0.34^*∗∗*^
C_max_/mg^.^L^−1^	4.84 ± 1.89	10.48 ± 0.63^*∗∗*^	5.83 ± 1.53	9.04 ± 3.04^*∗*^	10.49 ± 1.24^*∗∗*^

Note: compared with the control group, ^*∗*^*P* < 0.05; ^*∗∗*^*P* < 0.01.

**Table 2:** Effect of *P. ginseng* on the pharmacokinetics of caffeine on day 7 after administration.

Parameters/unit	Control	*P. ginseng*-L	*P. ginseng*-M	*P. ginseng*-H
AUC_(0-t)_/mg^.^L^-1.^h	2.28 ± 0.62	6.44 ± 2.32^*∗∗*^	8.20 ± 1.41^*∗∗*^	3.54 ± 0.84^*∗*^
AUC_(0-*∞*)_/mg^.^L^-1.^h	2.62 ± 0.55	7.07 ± 2.35^*∗∗*^	11.99 ± 3.16^*∗∗*^	3.86 ± 0.81^*∗*^
t_1/2z_/h	0.48 ± 0.31	0.60 ± 0.21	1.06 ± 0.36	0.46 ± 0.13
T_max_/mg^.^L^−1^	0.28 ± 0.19	0.11 ± 0.07	0.08 ± 0.00^*∗*^	0.17 ± 0.09
CL_z_/L^.^h^-1.^kg^−1^	7.94 ± 1.76	0.39 ± 0.15^*∗∗*^	0.22 ± 0.06^*∗∗*^	0.68 ± 0.18^*∗∗*^
C_max_/mg^.^L^−1^	3.04 ± 0.29	7.49 ± 2.56^*∗∗*^	10.38 ± 2.43^*∗∗*^	4.56 ± 1.14^*∗∗*^

Note: compared with the control group, ^*∗*^*P* < 0.05; ^*∗∗*^*P* < 0.01.

**Table 3:** Effect of *P. ginseng* on the pharmacokinetics of tolbutamide on day 7 after administration.

Parameters/unit	Control	*P. ginseng*-L	*P. ginseng*-M	*P. ginseng*-H
AUC_(0-t)_/mg^.^L^-1.^h	263.69 ± 21.75	236.95 ± 30.48	259.24 ± 33.53	287.59 ± 52.61
AUC_(0-*∞*)_/mg^.^L^-1.^h	273.05 ± 25.6	265.70 ± 28.38	288.31 ± 35.17	314.43 ± 59.49*∗*
t_1/2z_/h	4.86 ± 0.80	7.71 ± 2.71	7.59 ± 2.24	6.93 ± 1.00
T_max_/mg^.^L^−1^	0.42 ± 0.46	0.08 ± 0.00	0.08 ± 0.00	0.24 ± 0.15
CL_z_/L^.^h^-1.^kg^−1^	0.02 ± 0.00	0.02 ± 0.00	0.02 ± 0.00	0.02 ± 0.00
C_max_/mg^.^L^−1^	38.71 ± 5.56	38.76 ± 9.51	55.91 ± 7.86*∗∗*	42.96 ± 7.88

Note: compared with the control group, ^*∗*^*P* < 0.05; ^*∗∗*^*P* < 0.01.

**Table 4:** Effect of *P. ginseng* on the pharmacokinetics of chlorzoxazone on day 7 after administration.

Parameters/unit	Control	*P. ginseng*-L	*P. ginseng*-M	*P. ginseng*-H
AUC_(0-t)_/mg^.^L^-1.^h	13.53 ± 2.42	9.37 ± 1.93	19.05 ± 4.28^*∗*^	16.72 ± 5.96
AUC_(0-*∞*)_/mg^.^L^-1.^h	20.50 ± 6.59	11.89 ± 3.80^*∗*^	20.45 ± 5.10	18.39 ± 5.55
t_1/2z_/h	0.59 ± 0.25	0.40 ± 0.24	0.47 ± 0.11	0.30 ± 0.08^*∗*^
T_max_/mg^.^L^−1^	0.11 ± 0.07	0.08 ± 0.00	0.08 ± 0.00	0.17 ± 0.09
CL_z_/L^.^h^-1.^kg^−1^	1.07 ± 0.37	1.85 ± 0.64^*∗*^	1.02 ± 0.21	1.17 ± 0.34
C_max_/mg^.^L^−1^	21.32 ± 2.54	14.79 ± 2.28^*∗∗*^	28.51 ± 4.92^*∗∗*^	25.08 ± 6.49

Note: compared with the control group, ^*∗*^*P* < 0.05; ^*∗∗*^*P* < 0.01.

## Data Availability

The data used to support the findings of this study are available from the corresponding author upon request.

## Abbreviations

PXR: Pregnane X receptor
CYP450: Cytochrome P450
HLMs: Human liver microsomes
TCM: The traditional Chinese medicine
AUC: The area under the curves
MRT: Mean retention time
Cmax: Maximum concentrations
t_1/2_: Elimination half-lives
CL: The clearance
V: Apparent volume of distribution
ANF: Alpha-naphthoflavone
SPE: Sulfaphenazole
KEN: Ketoconazole
DDTC: Diethyldithiocarbonate.

## Supplementary Materials

Supplementary Materials Table S1: prime sequences for Q-PCR. Table S2: standard curves, linear range, and correlation coefficient of aconitine, mesaconitine, and hypaconitine in microsome. Table S3: standard curves, linear range, and correlation coefficient of probe drugs in rat plasma. Table S4: intra- and interday precision and accuracy of these standard curves. Table S5-1: the stability at 4°C and room temperature of detected methods. Table S5-2: the stability at freeze-thaw of detected methods. Table S6: recovery of the developed methods. Figure S1: the chromatograms of various probe drugs in plasma samples. 1: diazepam (IS); 2: caffeine; 3: tolbutamide; 4: chlorzoxazone; 5: midazolam.
