# Supplementary material for: Panax ginseng Inhibits Metabolism of Diester Alkaloids by Downregulating CYP3A4 Enzyme Activity via the Pregnane X Receptor
Source: Evid Based Complement Alternat Med. 2019 Mar 21;2019:3508658. doi: 10.1155/2019/3508658 (PMC6463675; doi:10.1155/2019/3508658)
Supplement: Supplementary Materials — Table S1: prime sequences for Q-PCR. Table S2: standard curves, linear range, and correlation coefficient of aconitine, mesaconitine, and hypaconitine in microsome. Table S3: standard curves, linear range, and correlation coefficient of probe drugs in rat plasma. Table S4: intra- and interday precision and accuracy of these standard curves. Table S5-1: the stability at 4°C and room temperature of detected methods. Table S5-2: the stability at freeze-thaw of detected methods. Table S6: recovery of the developed methods. Figure S1: the chromatograms of various probe drugs in plasma samples. 1: diazepam (IS); 2: caffeine; 3: tolbutamide; 4: chlorzoxazone; 5: midazolam. [file 3508658.f1.doc]

**Table S1. Prime sequences for Q-PCR.**

| Species | Prime | 5'-3' | Sequences |
| --- | --- | --- | --- |
| Rats | GAPDH | forward | GGCATCGTGGAAGGGCTC |
|  | GAPDH | reverse | GACCTTGCCCACAGCCTT |
|  | CYP3A2 | forward | AGATGCCTTTAGGTCCAATGGG |
|  | CYP3A2 | reverse | GCTGGAGATAGCAATGTTCGT |
|  | PXR | forward | CGAGCTCCGCAGCATCA |
|  | PXR | reverse | TGTATGTCCTGGATGCGCA |


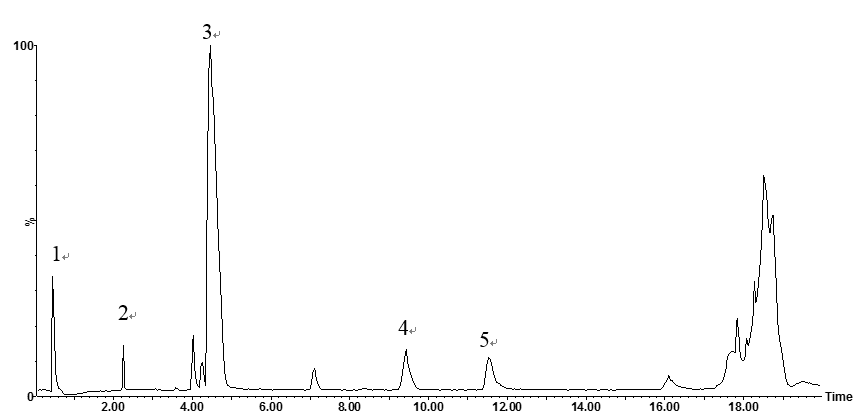


Fig.S1.The chromatograms of various probe drugs in plasma samples.

1: Diazepam (IS); 2:Caffeine; 3:Tolbutamide; 4:Chlorzoxazone; 5: Midazolam.

**Table S2.** Standard curves, linear range, and correlation coefficient of aconitine, mesaconitine and hypaconitine in microsome.

| Compounds | Standard curves | Linear range (µg/ml) | R^2^ |
| --- | --- | --- | --- |
| Aconitine | y=11.703x+16.12 | 5-160 | 0.9948 |
| Mesaconitine | y=8.1798x+14.451 | 5-160 | 0.9920 |
| Hypaconitine | y=8.8818x+18.378 | 5-160 | 0.9985 |

Note: To investigate whether the metabolism of aconitine, mesaconitine, and hypaconitine was affected by P. ginseng, the contents of aconitine, mesaconitine, and hypaconitine were determined in the incubation system after exposure to P. ginseng.

**Table S3.** Standard curves, linear range, and correlation coefficient of probe drugs in rat plasma.

| Probe | Standard curves | Linear range (µg/ml) | R^2^ |
| --- | --- | --- | --- |
| Caffeine | y=0.0104x+0.0162 | 0.03-64 | 0.9976 |
| Midazolam | y=0.1179x+0.0445 | 0.0075-16 | 0.9920 |
| Tolbutamide | y=0.0111x+0.0055 | 2-64 | 0.9984 |
| Chlorzoxazone | y=0.0072x+0.0104 | 0.03-64 | 0.9948 |

Note: The cocktail method was used to evaluate the effects of P. ginseng on CYP isoform activities in rats. The probe drugs, caffeine, midazolam, tolbutamide, and chlorzoxazone, were represent CYP1A2, CYP3A4, CYP2C9, and CYP2E1 respectively.

**Table S4.**  Intra and Inter-day precision and accuracy of these standard curves.

| Probe | QC conc.  (µg/ml) | Intra-day | | | Inter-day | | |
| --- | --- | --- | --- | --- | --- | --- | --- |
|  |  | Concentration (µg/ml) | RSD (%) | Accuracy (%) | Concentration (µg/ml) | RSD (%) | Accuracy (%) |
| Caffeine | 0.10 | 0.11±0.01 | 6.27 | 110.96±6.96 | 0.10±0.01 | 5.82 | 101.88±5.93 |
|  | 5.00 | 5.05±0.09 | 1.76 | 101.02±1.77 | 5.03±0.08 | 1.67 | 100.65±1.69 |
|  | 50.00 | 51.04±0.66 | 1.33 | 102.07±1.35 | 50.47±0.83 | 1.64 | 100.93±1.65 |
| Midazolam | 0.02 | 0.02±0.00 | 2.60 | 100.82±2.62 | 0.02±0.00 | 4.10 | 97.60±4.00 |
|  | 0.12 | 0.12±0.00 | 2.57 | 101.72±2.61 | 0.12±0.00 | 1.39 | 100.27±1.40 |
|  | 12.00 | 11.97±0.11 | 0.94 | 99.72±0.93 | 11.98±0.11 | 0.88 | 99.84±0.88 |
| Tolbutamide | 5.00 | 4.93±0.18 | 3.67 | 98.65±3.64 | 5.01±0.09 | 1.70 | 100.16±1.71 |
|  | 25.00 | 24.67±0.55 | 2.24 | 98.68±2.21 | 24.86±0.31 | 1.24 | 99.43±1.23 |
|  | 50.00 | 50.05±0.53 | 1.06 | 100.09±1.06 | 49.92±0.42 | 0.84 | 99.84±0.84 |
| Chlorzoxazone | 0.10 | 0.10±0.01 | 5.58 | 101.86±5.68 | 0.10±0.00 | 3.65 | 103.33±3.77 |
|  | 5.00 | 5.00±0.07 | 1.49 | 99.95±1.49 | 4.97±0.08 | 1.65 | 99.34±1.63 |
|  | 50.00 | 50.02±0.67 | 1.35 | 100.04±1.35 | 50.50±0.84 | 1.66 | 101.00±1.67 |

Note: The cocktail method was used to evaluate the effects of P. ginseng on CYP isoform activities in rats. The probe drugs, caffeine, midazolam, tolbutamide, and chlorzoxazone, were represent CYP1A2, CYP3A4, CYP2C9, and CYP2E1 respectively. The average intra-day and inter-day precision and accuracy for the high, medium, and low concentrations ranged from 97.60% to 110.96%. The RSDs of intra-day and inter-day precision and accuracy were less than 10%.

**Table S5-1** The stability at 4℃ and room temperature of detected methods.

| Probe | QC conc.  (µg/ml) | 4℃ temperature stability (24h) | | | Room temperature stability (24h) | | |
| --- | --- | --- | --- | --- | --- | --- | --- |
|  |  | Concentration (µg/ml) | RSD (%) | Accuracy (%) | Concentration (µg/ml) | RSD (%) | Accuracy  (%) |
| Caffeine | 0.10 | 0.10±0.01 | 8.12 | 103.70±8.42 | 0.11±0.01 | 12.24 | 107.25±13.13 |
|  | 5.00 | 5.03±0.34 | 6.77 | 100.51±6.81 | 4.82±0.13 | 2.60 | 96.42±2.50 |
|  | 50.00 | 51.34±0.75 | 1.47 | 102.68±1.51 | 48.38±1.32 | 2.72 | 96.76±2.64 |
| Midazolam | 0.02 | 0.02±0.00 | 4.11 | 93.69±3.86 | 0.02±0.00 | 4.54 | 91.06±4.13 |
|  | 0.12 | 0.12±0.01 | 8.68 | 101.80±8.84 | 0.12±0.01 | 4.64 | 98.67±4.58 |
|  | 12.00 | 11.50±0.73 | 6.36 | 95.81±6.10 | 11.57±0.60 | 5.19 | 96.39±5.00 |
| Tolbutamide | 5.00 | 5.13±0.15 | 2.89 | 102.54±2.96 | 4.86±0.17 | 3.47 | 97.26±3.38 |
|  | 25.00 | 25.39±1.37 | 5.39 | 101.54±5.48 | 24.22±1.14 | 4.72 | 96.87±4.57 |
|  | 50.00 | 50.81±2.56 | 5.03 | 101.62±5.12 | 49.03±1.97 | 4.01 | 98.06±3.93 |
| Chlorzoxazone | 0.10 | 5.01±0.24 | 7.78 | 104.33±8.12 | 4.96±0.18 | 8.83 | 94.25±8.33 |
|  | 5.00 | 51.79±1.15 | 4.83 | 100.14±4.83 | 4.96±0.18 | 3.64 | 99.11±3.61 |
|  | 50.00 | 0.09±0.01 | 2.23 | 103.58±2.31 | 50.53±1.33 | 2.63 | 101.07±2.66 |

Note: The cocktail method was used to evaluate the effects of P. ginseng on CYP isoform activities in rats. The stability at 4℃ and room temperature of detected methods for four probe drugs were from 93.69±3.86% to 107.25±13.13%.

.

**Table S5-2** The stability at freeze-thaw of detected methods.

| Probe | QC conc.  (µg/ml) | Freeze-thaw | | |
| --- | --- | --- | --- | --- |
|  |  | Concentration (µg/ml) | RSD (%) | Accuracy (%) |
| Caffeine | 0.10 | 0.10±0.01 | 8.95 | 102.63±9.18 |
|  | 5.00 | 5.04±0.21 | 4.06 | 100.79±4.09 |
|  | 50.00 | 51.08±1.56 | 3.04 | 102.16±3.12 |
| Midazolam | 0.02 | 0.02±0.00 | 3.27 | 91.76±3.00 |
|  | 0.12 | 0.11±0.01 | 6.10 | 94.65±5.78 |
|  | 12.00 | 11.37±0.51 | 4.51 | 94.77±4.28 |
| Tolbutamide | 5.00 | 4.78±0.21 | 4.39 | 95.53±4.20 |
|  | 25.00 | 24.01±2.07 | 8.62 | 96.05±8.28 |
|  | 50.00 | 49.68±2.60 | 5.23 | 99.34±5.19 |
| Chlorzoxazone | 0.10 | 0.091±0.01 | 7.20 | 90.88±6.54 |
|  | 5.00 | 4.85±0.21 | 4.28 | 97.02±4.16 |
|  | 50.00 | 48.06±2.55 | 5.30 | 96.12±5.10 |

Note: The cocktail method was used to evaluate the effects of P. ginseng on CYP isoform activities in rats. The stability at freeze-thaw of detected methods for four probe drugs were from 90.88±6.54% to102.63±9.18 %.

**Table S6** Recovery of the developed methods.

| Probe | QC conc. (µg/ml) | Recovery (mean±SD) | RSD (%) |
| --- | --- | --- | --- |
| Caffeine | 0.10 | 97.17±9.46 | 9.73 |
|  | 5.00 | 93.01±6.48 | 6.97 |
|  | 50.00 | 91.41±8.61 | 9.42 |
| Midazolam | 0.02 | 102.00±10.57 | 10.36 |
|  | 0.12 | 99.64±2.60 | 2.61 |
|  | 12.00 | 101.08±2.51 | 2.48 |
| Tolbutamide | 5.00 | 97.34±4.19 | 4.30 |
|  | 25.00 | 98.82±9.91 | 10.03 |
|  | 50.00 | 102.11±6.89 | 6.75 |
| Chlorzoxazone | 0.10 | 100.89±9.69 | 9.60 |
|  | 5.00 | 96.89±5.18 | 5.35 |
|  | 50.00 | 98.30±5.60 | 5.70 |

Note: The cocktail method was used to evaluate the effects of P. ginseng on CYP isoform activities in rats. Recovery of the developed methods for four probe drugs were from 91.41±8.61% to 102.11±6.89 %.
